# Single-stage repair for acute type A aortic dissection complicated by rupture of the infrarenal aorta

**DOI:** 10.1016/j.xjse.2024.100021

**Published:** 2024-08-29

**Authors:** Gabriele Piffaretti, Maria Cristina Cervarolo, Paolo Borsani, Federico Fontana

**Affiliations:** a Division of Vascular Surgery, Department of Medicine and Surgery, University of Insubria School of Medicine, Varese, Italy; b Division of Cardiac Surgery, Department of Cardio-Thoracic and Vascular Surgery, ASST Settelaghi Circolo University Hospital, Varese, Italy; c Interventional Radiology, Department of Medicine and Surgery, University of Insubria School of Medicine, Varese, Italy

**Figure:**
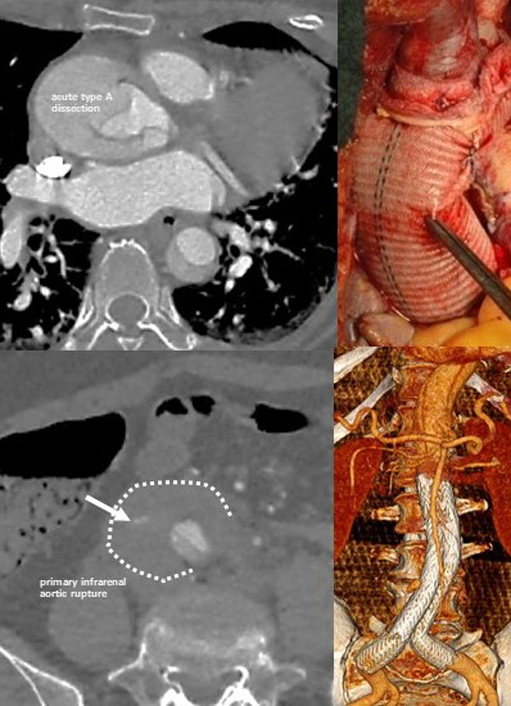
Simultaneous repair for concomitant type A dissection and infrarenal aortic rupture.

**Central Message**

Acute type A aortic dissection complicated by abdominal rupture is a rare event with no clear recommendation as far as surgical strategy is concerned when these 2 entities present simultaneously.

Overall mortality of acute type A aortic dissection (ATAAD) is significantly higher in complicated cases, especially when patients present with malperfusion syndromes.^1^ Among vascular complication of ATAAD, no article has precisely described the site of rupture and its correlation with perioperative outcomes.^2^ Few case reports specifically describe rupture in an already enlarged abdominal aorta simultaneous to ATAAD, and guidelines do not describe alternative strategies when such life-threatening lesions present simultaneously.^1^^,^^3^, ^4^, ^5^ We report the case of a single-stage repair of an ATAAD complicated by primary rupture of a nondilated infrarenal aorta and peripheral malperfusion.

## Case Description

**Figure 1:**
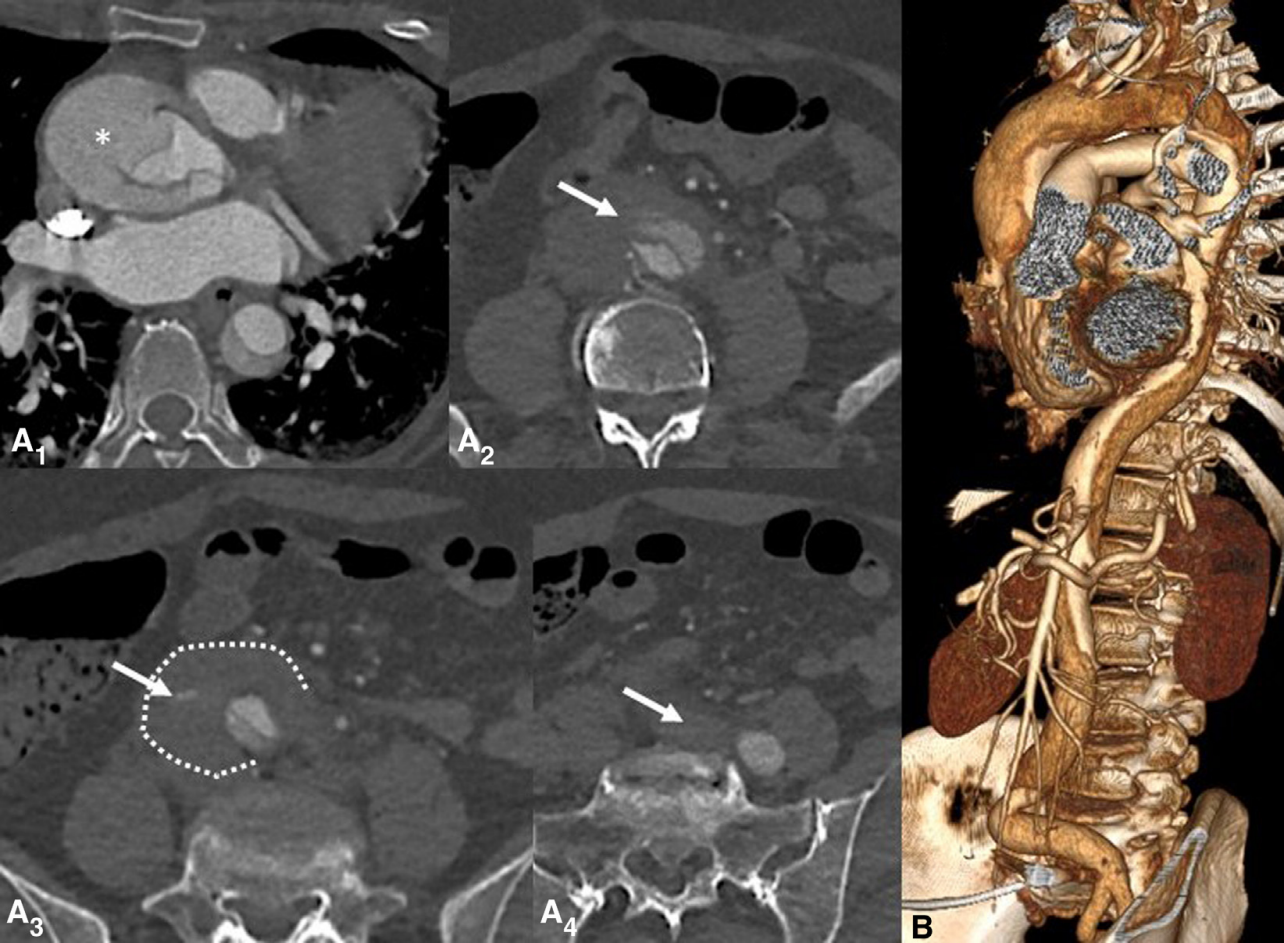
Preoperative computed tomography angiography showing acute type A aortic dissection (A_1_) (*asterisk*) with the infrarenal rupture (A_2_) (*white arrow*) that was contained with a recent bleeding inside (A_3_) (*white arrow*) the retroperitoneal hematoma (A_3_) (*dotted line*). Peripheral malperfusion was caused by static obstruction of the right common iliac artery (A_4_) (*white arrow*). Three-dimensional volume rendering reconstruction of the Debakey type 1 dissection (B).

**Figure 2:**
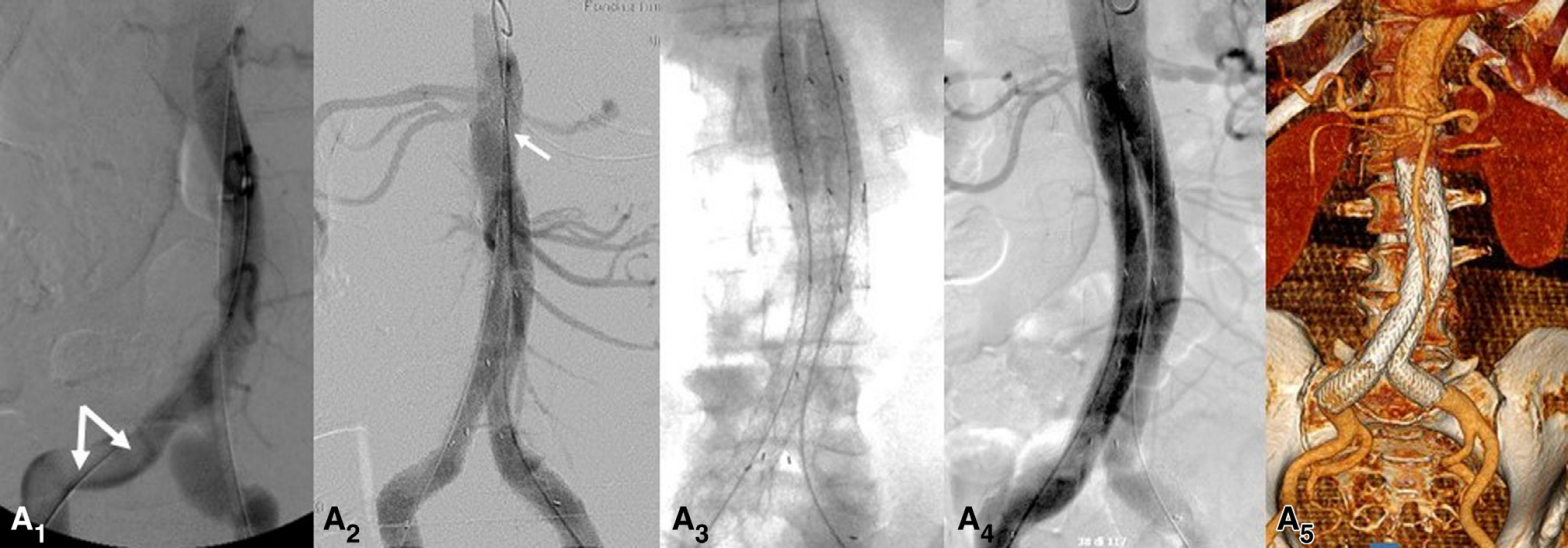
Intraoperative findings of endovascular repair of the infrarenal aorta. Preliminary angiography showing the dissecting flap (A_1_) (*white arrows*). Postendograft deployment control which showed the presence of an incomplete apposition of the endograft (A_2_) (*white arrow*). Reinforcement of the proximal edge of the endograft with kissing balloon expandable stent-grafting (A_3_). Final control with complete relamination of the lamella at the level of the renal arteries (A_4_). Six-month follow-up computed tomography angiography (A_5_).

A 57-year old female patient, a heavy smoker with a silent medical history, was transferred to the emergency department of our academic hospital soon after the abrupt onset of chest pain. Preoperative computed tomography angiography revealed a Debakey type 1 ATAAD with an enlarged (47 mm) ascending aorta complicated by infrarenal contained rupture of a nondilated aorta, and peripheral malperfusion due to the static occlusion of the right common iliac artery (Figure 1, *A*_*1-4*_ and *B*). She was prepared for a single-stage approach by 2 surgical teams (ie, cardiac and vascular). Considering the absence of active bleeding and the rapid worsening of her aortic valve insufficiency, we opted to start with proximal aortic graft replacement. Through right axillary artery cannulation and median sternotomy, hypothermic circulatory arrest (30 °C) with antegrade cerebral perfusion were instituted, and ascending graft replacement (Hemashield Platinum 30 mm; Boston Scientific) was performed along with valve repair with commissure resuspension. The retroperitoneal hematoma did not expand with full heparinization during ATAAD repair. Immediately after open repair, endovascular repair of the infrarenal aorta was performed with a bifurcated endograft (EG) (C3 Exclude 23 mm; W.L. Gore & Associates) reinforced with additional kissing balloon expandable stent-grafting (VBX; W.L. Gore & Associates) to optimize the relamination at the level of the renal arteries (Figure 2, *A*_*1-4*_). Discharged on postoperative day 20, she was still alive at 6-months when follow-up computed tomography angiography confirmed the complete exclusion of the abdominal rupture and the absence of dissection-related complications. Consent for publication was not required by the local institutional review board according to the Italian National Policy Privacy Act on retrospective analysis of anonymized data.

## Discussion

Rupture of the infrarenal aorta has been reported to be a potential complication of ATAAD: in their study on vascular complications associated with spontaneous dissections, Cambria and colleagues^2^ reported an incidence of infrarenal rupture of 0.3% out of 325 lesions. However, the real incidence of this life-threatening complication is unknown because it has been poorly reported in the literature. This is the main reason why recent professional cardiovascular guidelines have not specifically addressed the optimal strategy in such a challenging scenario.^1^

The most difficult aspect in this circumstance is correlated to the type of strategy to be adopted, as well as the timing of the repair: Should ATAAD be addressed first with the potentially fatal bleeding in the abdomen, or should repairing the infrarenal-contained rupture happen first, which may leave operators with the high risk of sudden death as a result of the unfixed ATAAD? In contrast to malperfusion syndromes, the absence of robust data from the literature has left the most recent guidelines deprived of helpful indications for this rare but challenging scenario.^1^ In our case, the rapid worsening of aortic valve insufficiency led us to resolve the proximal aortic repair first; we relied on the low-pressure regimen of the circulatory arrest to limit the risk that an infrarenal aortic-contained rupture would eventually progress toward a free rupture. Few articles report operative repair of these synchronous lesions; generally, infrarenal rupture occurs on an already dilated aorta or iliac artery, and open aorto-iliac graft replacement has been the mainstay of treatment.^3^, ^4^, ^5^ From a technical point of view, previous experience dictated that occluding a re-entry by abdominal aortic repair can lead to formation of a juxtarenal blind-ending false lumen.^4^ Because no arch tear was observed, total arch replacement was not performed; therefore, a complete thoracoabdominal relamination of the lamella would have not been possible. In contrast, we believed that reinforcing the EG with a kissing balloon-stent grafting would have promoted the lamina remodeling exactly at the site of the proximal landing zone, thus reducing the extent of the aortic repair but also mitigating the risk of a cul de sac type of false lumen in proximity of the EG.

## Conclusions

Primary rupture of a nondilated infrarenal aorta complicating ATAAD is a rare event. Simultaneous repair should be contemplated, and endovascular repair may be effective by sealing the rupture and promoting relamination at the proximal landing zone.

## Conflict of Interest Statement

The authors reported no conflicts of interest.

The *Journal* policy requires editors and reviewers to disclose conflicts of interest and to decline handing or reviewing manuscripts for which they may have a conflict of interest. The editors and reviewers of this article have no conflicts of interest.
